# Electrically-switched differential microscopy based on computing liquid-crystal platforms

**DOI:** 10.1515/nanoph-2023-0688

**Published:** 2024-01-24

**Authors:** Shuoqing Liu, Dandan Zheng, Qiang Yang, Shizhen Chen, Shuangchun Wen, Hailu Luo

**Affiliations:** Key Laboratory of Micro-/Nano-Optoelectronic Devices of Ministry of Education and Hunan Provincial Key Laboratory of Low-Dimensional Structural Physics and Devices, School of Physics and Electronics, Hunan University, Changsha 410082, China

**Keywords:** computing liquid-crystal, all-optical image processing, optical differential operation, edge-enhanced imaging, microscopy imaging

## Abstract

Detection of transparent phase specimens especially biological cells with desired contrasts is of great importance in visual display and medical diagnosis. Due to the pure-phase nature, conventional detection approaches may damage samples or require complex operations. Computing liquid crystal (LC) is a thin and flat optical element with excellent capability in optical field modulation, which gives a feasible way to this issue from the perspective of analog optical computing. We here propose and experimentally implement an electrically switched two-dimensional (2D) differential microscopy based on computing LC platforms. The Pancharatnam–Berry phase LC polarization grating induces light’s spin separation to promote the 2D differential operation. Using the electrically tunable LC plate as the system phase retardance provider, the detecting mode can be flexibly switched from bright-field images to edge-enhanced images with desired contrasts. Remarkably, owing to the wavelength-independent feature closely related to the geometric phases, our proposed scheme is demonstrated to be applicable to the multi-wavelength microscopy imaging. These results open avenues to form real-time all-optical image processing and may facilitate multifunctional differential microscopy.

## 1 Introduction

Visualization of biological cells and tissues is a fundamental but key issue in biological research and medical diagnosis [1], [2], [3], [4], [5], [6], [7]. In general, cells with significant light scattering intensity or thickness, which can be treated as amplitude objects, are easy for observation. However, for transparent biological samples such as single cells and thin tissue sections, since no apparent absorption appears in visible spectrums, the intensity of scattered light is much weaker than the incident one. Meanwhile, due to the ultra-thin structures, this class of specimens only has distinct influences on the wavefront phase but not on its amplitude, and they are referred as the phase objects, greatly limiting the realization of high-contrast phase imaging [4], [7]. To enable such structures visible, one common approach is to stain the phase object by fluorescent labeling to enhance imaging resolution [8], [9]. However, this method may destroy the sample and even alter the normal physiology of the cell inevitably. Other phase detecting methods, such as phase-contrast and differential-interference-contrast microscopes, can realize a label-free visualization [1], [2], but they often require complex operations with bulky components or may result in false contrast due to ambient halo. Besides, these methods are often restricted to one-dimensional (1D) imaging.

Recently, an emergent signal processing technique called analog optical computing has attracted wide attentions [10], [11], [12]. Such an approach can perform the image processing with ultra-fast speed and extremely low energy consumption, as well as large-scale operation parallelism. The optical differentiation and solving integration are typical aspects of analog optical computing. In particular, the image differentiation for edge-based enhancement offers possibilities for numerous applications in biological imaging and computer vision [12], [13]. From this perspective, some real-time and label-free phase imaging schemes without destroying samples can be achieved based on spatial differentiation [14], [15], [16], [17], [18]. For example, the interference effects associated with surface plasmon excitations at a single metal–dielectric interface [14] and the photonic spin Hall effect at a planar interface [15], [16], [17], [18]. By adjusting the post-selected state, pure phase gradient can be extracted individually from the mixture [19]. Remarkably, applying emerging geometric phase optical components, several multifunctional devices with reduced scale have been designed to satisfy wide applications such as nanoprinting [20], spatial phase modulator and polarization controller [21], [22], [23], [24], [25], [26], multifunctional optical differentiator [27], [28], [29], [30], [31], [32], [33], [34], and even phase imaging [35], [36], [37]. However, some of these schemes require additional bulky elements or high fabrication cost without dynamic tunability and are difficult to accomplish broadband image processing.

Liquid crystal (LC) is a kind of flat optical element with high optical efficiency by artificially designing periodic structures, where the LC molecules with anisotropic shapes are often self-assemble for modulating the geometric phases flexibly [38], [39]. Therefore, it is believed that LC can provide an effective way to tackle the existing challenging issues. As an emerging computing platform, LC can adjust the polarization and phase of photons for desired applications by conferring specific LC molecular arrangements. For example, serving as an LC cell with disordered initial state of molecules, it can convert into a switchable phase plate (PP) with modulated external voltages, so as to construct a phase retardance device adapted to the needs of practical scenarios [40], [41]. If treated as a polarization grating (PG) with regular molecular arrangements, the proper geometric phase of LC naturally induces tiny spin-dependent separations, contributing to the mathematical operation of spatial differentiation for image edge detection [41], [42], [43], [44]. Meanwhile, the unique fabrication of LC by photo-alignment technique endues itself with significant optical properties, such as high transmission, small volume, and wavelength independent [45]. These studies also make the LCs excellent candidates for realizing the real-time all-optical image processing in multiple wavelengths. Although the LCs have exhibited the excellent capabilities for image processing, to our best knowledge, the two-dimensional (2D) microimaging of biological cells with flexible edge-enhancement operations in multi-wavelength has not been discussed and experimentally achieved.

In this paper, we propose a multi-functional differential microscopy exploiting electrically switched computing LCs toward the high-performance visualization of biological cells and tissues. To extract clear 2D edge profiles of pure phase objects, we design two mutually orthogonal paths of analog optical computing based on the LC PG-induced spin-dependent separations. As a result, the differential operations along the *x*-direction and the *y*-direction can be realized simultaneously, and the 2D image edge can be accessed in real-time. By introducing the electrically switched LC PP with phase retardance precisely modulated by the applied voltage, we can rapidly switch the edge-enhanced images of the phase object for desired contrasts. Combined with optical microscopes, our scheme can achieve the visualization of pure quantitative phase microscopy targets and transparent biological samples under multi-wavelength conditions. These results not only bridge the analog optical computing and the phase imaging based on the computing LCs but also expand the pathway for the realization of multifunctional dark-field differential microscopy.

## 2 Principle and design of an electrically-switched differential operation platform

**Figure 1:**
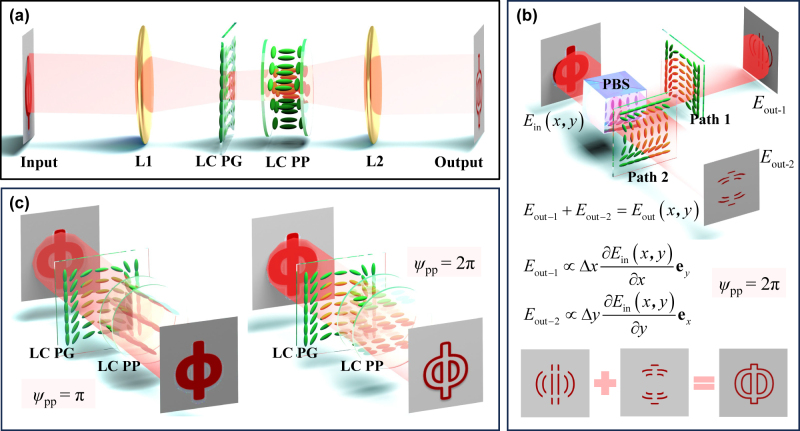
Schematic of the electrically switched optical differentiator endowed by the computing liquid-crystal platform. (a) Illustration of the differential operation by the platform composed of a computing liquid crystal (LC) polarization grating (PG) and an electrically switched LC phase plate (PP). (b) The direct implementation of the two-dimensional (2D) differential operation under phase retardance *ψ*_PP_ = 2*π*. The differential results in both *x*- and *y*-directions are acquired simultaneously. (c) Switching of the output mode from the bright-field image to the edge image when the phase retardance is modulated from *ψ*_PP_ = *π* to *ψ*_PP_ = 2*π*.

We design an electrically switched differential operation system driven by a computing liquid-crystal platform, as illustrated in Figure 1(a). To realize the proposed function, a 4f system formed by two lenses (L) is utilized in conjunction, and a static LC PG and an electrically tuned LC PP are inserted near the Fourier plane of the 4f system, along with a pair of crossed polarizers. The LC PG resembles a half-wave plate with anisotropic periodic index profiles, which possesses a unified phase retardation of *ψ*_PG_ = *π* and spatially varying LC molecules in direction of *ρ*_PG_. The LC PP can be regarded as a waveplate with an electrically switched phase retardance *ψ*_PP_ in optical axis direction of *ρ*_PP_ = *π*/4, and additional phase retardances can be provided by precisely applying the external voltage for the system. Such elements can be regarded as excellent candidates in optical field modulation and operation. When the input image is processed by this platform, the output edge-enhanced images can be presented retaining important initial geometric features. This process can be regarded as the performance of an electrically switched differentiator.

To explain the concept of proposed differentiator, we subsequently discuss the operation from the bright-field to the differentiation, as schematically shown in Figure 1(b) and (c). To acquire the complete 2D profile, two optical paths need to be taken into account (see Figure 1(b)). For path 1, consider the incident linearly polarized (LP) light along the *x*-direction, i.e., with horizontal (*H*) polarization, and the electric field is distributed as

(1) $$E_{\text{in}}\left(x,y\right)=E_{\text{in}}\left(x,y\right)e_{x}\propto E_{\text{in}}\left(x,y\right)\left(e_{+}+e_{-}\right).$$

Here, **e**_+_ and **e**_−_ denote the electric-field vectors of left-handed circular polarized (LCP) and right-handed circular polarized (RCP) components. After passing through the LC PG (optical axes vary along the *x*-direction), the circular polarizations reverse their handedness, and a spatially varying Pancharatnam–Berry (PB) phase *φ*_PB_ = −2*σ*_±_ *ρ*_PG_ is induced in the real space [19], [43], [46]. Here, *σ*_+_ = +1 and *σ*_−_ = −1 correspond to the LCP and RCP components, respectively. In this process, the PB phase gradient manifests itself as the spin-dependent shift in the momentum space $\Delta k_{x}=\left(\partial \varphi_{\text{PB}}/\partial x\right)e_{x}=-\sigma_{\pm }2\pi /d$ . Converting the coordinate into real space after a propagation distance *z*, the momentum shift will result in a real-space shift $\Delta x=\left({\Delta k}_{x}/k_{0}\right)z=\mp \lambda z/d$ , where *k*_0_ and *λ* denote the wave number and the wavelength of incidence correspondingly. Therefore, the electric field distribution evolves as $E_{\text{PG}}\left(x,y\right)=E_{\text{in}}\left(x+\Delta x,y\right){\left(1\;i\right)}^{T}+E_{\text{in}}\left(x-\Delta x,y\right){\left(1\;-i\right)}^{T}$ . Consider that the PB phase gradient is small, Δ*x* is tiny in comparison with the field distribution, and the expression can be further approximated as $E_{\text{PG}}\left(x,y\right)≃2E_{\text{in}}\left(x,y\right)e_{x}+\left[i2\Delta x\partial E_{\text{in}}\left(x,y\right)/\partial x\right]e_{y}$ [43]. After passing through the LC PP, the field is formed as

(2) $$\begin{array}{cc} E_{\text{PP}}\left(x,y\right) & ≃\left[-iK_{1}E_{\text{in}}\left(x,y\right)+iK_{2}\Delta x\frac{\partial E_{\text{in}}\left(x,y\right)}{\partial x}\right]e_{y}\\ & +\left[K_{2}E_{\text{in}}\left(x,y\right)+K_{1}\Delta x\frac{\partial E_{\text{in}}\left(x,y\right)}{\partial x}\right]e_{x}. \end{array}$$

Here, $K_{1}=2\sin\left(\psi_{\text{PP}}/2\right)$ and $K_{2}=2\cos\left(\psi_{\text{PP}}/2\right)$ , and a phase retardance *ψ*_PP_ is endowed to modulate the allocation of bright-field images and 1D differential results along the *x*-direction. Subsequently, the light is only released in the *y*-direction, and the output field is given by

(3) $$E_{\text{out}-1}\left(x,y\right)=\left[-iK_{1}E_{\text{in}}\left(x,y\right)+iK_{2}\Delta x\frac{\partial E_{\text{in}}\left(x,y\right)}{\partial x}\right]e_{y}.$$

Similarly, for the incident LP light along the *y*-direction [with vertical (*V*) polarization], path 2 can realize the differential operation along the *y*-direction after the LC PG (optical axes vary along the *y*-direction) and the LC PP. And the output field is distributed as

(4) $$E_{\text{out}-2}\left(x,y\right)=\left[-iK_{1}E_{\text{in}}\left(x,y\right)+iK_{2}\Delta y\frac{\partial E_{\text{in}}\left(x,y\right)}{\partial y}\right]e_{x}$$

with a tiny spin-dependent separation Δ*y*. As a result, the final output field of the whole platform is given by

(5) $$\begin{array}{cc} E_{\text{out}}\left(x,y\right) & \propto -iK_{1}\left[E_{\text{in}}\left(x,y\right)e_{y}+E_{\text{in}}\left(x,y\right)e_{x}\right]\\ & +iK_{2}\left[\Delta x\frac{\partial E_{\text{in}}\left(x,y\right)}{\partial x}e_{y}+\Delta y\frac{\partial E_{\text{in}}\left(x,y\right)}{\partial y}e_{x}\right]. \end{array}$$

After setting *ψ*_PP_ = 2*π* (*K*_1_ = 0), the pure 2D image edge can be acquired as $E_{\text{out}}\left(x,y\right)\propto \left[\Delta x\partial E_{\text{in}}\left(x,y\right)/\partial x\right]e_{y}+\left[\Delta y\partial E_{\text{in}}\left(x,y\right)/\partial y\right]e_{x}$ by the superposition of differential results along the *x*- and *y*-directions (see Supplementary Material for details).

For the case of *ψ*_PP_ = *π* (*K*_2_ = 0), none of the second term of Eq. (5) is outputted, and the bright-field image is obtained. With *ψ*_PP_ varying from *π* to 2*π*, the output image is switched gradually from the bright-field to the differentiation, so that the edge signals (i.e., contrasts) can be enhanced artificially, as further presented in Figure 1(c). Therefore, our scheme can be applicable to 2D differential operation with electrically switched output mode by computing LCs.

Remarkably, the field after passing through the pure phase-contrast object such as transparent cells with phase distribution $\varphi \left(x,y\right)$ can be approximated as $E_{\text{in}}\left(x,y\right)=1\times {\text{e}}^{\text{i}\varphi \left(x,y\right)}$ , since the corresponding intensity satisfies $I_{\text{in}}\left(x,y\right)={\left|E_{\text{in}}\left(x,y\right)\right|}^{2}=1$ [7]. At this time, the output image only experiences a phase change. That is, from Eq. (5), the output field at *ψ*_PP_ = *π* (*K*_2_ = 0) is given by

(6) $$E_{\text{out}}\left(x,y\right)\propto {\text{e}}^{\text{i}\varphi \left(x,y\right)}e_{y}+{\text{e}}^{\text{i}\varphi \left(x,y\right)}e_{x},$$

which corresponds to the cooperative result of bright-field signals in both the *x*- and *y*-directions, and no contrast is shown in the output image. Distinctively, after the proposed differential operation with applying *ψ*_PP_ = 2*π* (*K*_1_ = 0), the output field is expressed by

(7) $$E_{\text{out}}\left(x,y\right)\propto {\text{e}}^{\text{i}\varphi \left(x,y\right)}\left[\Delta x\frac{\partial \varphi \left(x,y\right)}{\partial x}e_{y}+\Delta y\frac{\partial \varphi \left(x,y\right)}{\partial y}e_{x}\right].$$

As a result, the output intensity evolves as $I_{\text{out}}\left(x,y\right)\propto |\Delta x\partial \varphi \left(x,y\right)/\partial x{|}^{2}+|\Delta y\partial \varphi \left(x,y\right)/\partial y{|}^{2}$ (see Supplementary Material for details). To be specific, the phase gradient manifests itself as the intensity-contrast image (edge image) after the proposed operation platform, which makes it possible to realize the controllable dark-filed differential microscopy.

By the way, the optical mathematical operations and imaging methods based on 4f systems have been discussed for a long time, and our scheme here is to propose and implement technical improvements based on the previous ones. By exploiting the lightweight computing LCs that embody the polarization conversion and electrical modulation functions, we aim to provide an avenue to the flexible switching of operation modes and the direct extraction of the 2D edge profile of objects, without labeling the sample or performing complex experimental operations by bulky equipment. The related fundamentals are also applicable to the detection of micro-mechanisms and biological specimens, and may be extended to fields of face recognition, phase estimation, and other aspects.

## 3 Results and discussions

### 3.1 Fabrication and experimental features of computing LCs

**Figure 2:**
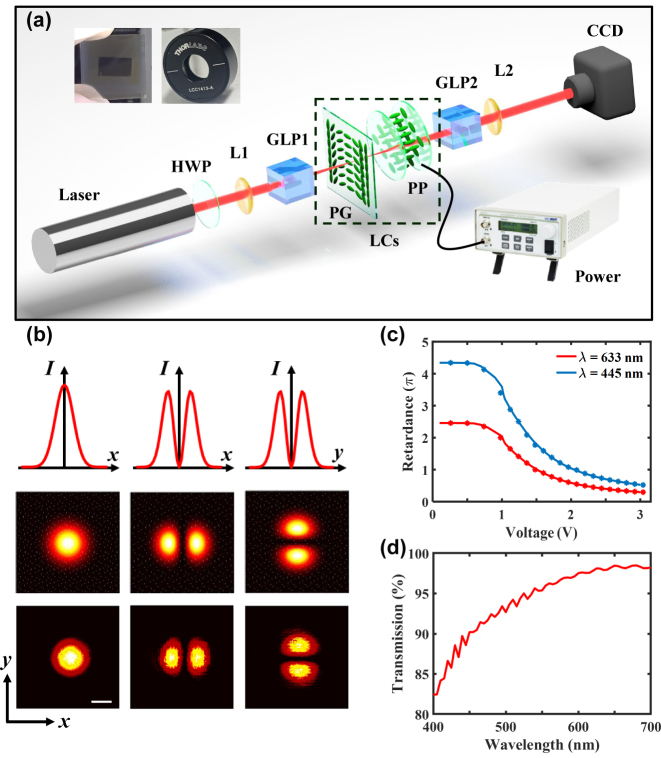
Experimental observation of the light intensity profile and the feature of LC elements. (a) Experimental setup. The laser generates the light beam with Gaussian distribution, and the half-wave plate (HWP) is adopted to modulate the light intensity. Lenses L1 and L2 focus and collimate the light (focal lengths *f*_1_ = *f*_2_ = 175 mm). GLP1 and GLP2 are a pair of mutually orthogonal Glan laser polarizers. The computing LCs consist of an LC PG and an LC PP driven by the power supply, and the insets show their photographs. The CCD camera captures the output intensity profile. (b) Comparison between the theoretical and the experimental intensity profiles at wavelength *λ* = 633 nm, including the input and output intensities after the LC PG. Scale bar: 500 μm. (c) Measured phase retardances of the LC PP with the variation of the applied voltage at wavelengths *λ* = 633 nm and *λ* = 445 nm, respectively. (d) Measured transmission of the LC PP with the variation of incident wavelength under applied voltage *u* = 0.98 V.

To demonstrate the differential operation of the proposed platform, we design the experimental setup to observe the light intensity profile and characterize the feature of LCs. The experimental setup is shown in Figure 2(a). The He–Ne laser (working wavelength *λ* = 633 nm, Thorlabs) provides the light source with Gaussian distribution, and the half-wave plate (HWP) is used to adjust the input intensity. Through the first Glan laser polarizer (GLP1), the LP beam for the input field is prepared, and GLP2 is applied to analyze the polarization of the output field after the computing LC elements. The two lenses (L1 and L2, focal lengths *f*_1_ = *f*_2_ = 175 mm) cooperate to constitute a 4f system, where the LC PG is placed at the confocal plane of L1 and L2. The charge-coupled device (CCD) is placed after L2 to capture the output intensity.

The top left insets show the photographs of LC PG and LC PP. The LC PG is fabricated on a 20 mm × 20 mm glass by the photo-alignment technology, and the effective working area is 10 mm × 6 mm with the diffraction angle of *θ* = 3.6°. Among it, the orientation of LC molecules varies along one direction (the *x*-direction and the *y*-direction) to form a periodically distribution $\rho_{\text{PG}}\left(x,y\right)=\pi x/d$ [or $\rho_{\text{PG}}\left(x,y\right)=\pi y/d$ ], and the corresponding period is formed by *d* = 10 mm. To fabricate this LC structure, we first spin-coat the sulfuric azo dye SD1 solution on the glass substrate and bake it under irradiation of polarized lights to form a photo-alignment film. Then, we coat a reactive mesogen mixture on this photo-alignment layer and expose it under a desired polarized field to ensure a stable and regular arrangement of LC molecules. The optical axes determined by the orientation of LC molecules are perpendicular to the exposure polarization [43]. After an LP beam is passed through such a designed PG, tiny spin separations are induced (Δ*k* in the momentum space and Δ*x* in the real-space), facilitating the optical differentiation. For the input light released by GLP1 in the *x*-direction (*H* polarization), its LCP and RCP components separate with each other along the *x*-direction. And the intensity pattern at this time consists of three parts, i.e., the LCP image, the RCP image, and the LP image maintaining the *H* polarization in the middle of their superposition. By introducing the analyzer (GLP2, with transmission axis orthogonal to GLP1), the middle image is filtered out and only the images on the sides are retained, which approximately corresponds to the image edge detection. The final captured intensity can be regarded as the differentiation of the input field along the *x*-direction, as shown in Figure 2(b). For the incidence in the *y*-direction (*V* polarization), the differential operation along the *y*-direction can also be realized. The experimental results satisfy well with the theoretical predictions, which demonstrate that our scheme is capable of performing spatial differential operation in the *x*- and *y*-directions. Note that from the perspective of image edge detection, the separation between the LCP and the RCP components determines how sharp the edge can be resolved, and the detection resolution can be given by *δ* = 2Δ*x* [33]. Since the period *d* can indeed influence Δ*x*, we may adjust the sharp of image edge and achieve a tunable detection resolution by carefully designing the period of LC PG.

It is also worth noting that the spatial spectral transfer function is given by $H\left(k_{x},k_{y}\right)={\tilde{E}}_{\text{out}}\left(k_{x},k_{y}\right)/{\tilde{E}}_{\text{in}}\left(k_{x},k_{y}\right)$ , where ${\tilde{E}}_{\text{out}}\left(k_{x},k_{y}\right)$ and ${\tilde{E}}_{\text{in}}\left(k_{x},k_{y}\right)$ are the Fourier transformations of electric fields $E_{\text{out}}\left(x,y\right)$ and $E_{\text{in}}\left(x,y\right)$ , respectively. Owing to the relation between the light intensity and the electric field, we can acquire $H\left(k_{x},k_{y}\right)$ by processing data extracted from the input and the output light fields (see Supplementary Material for details).

The adopted electrically switched LC PP can be seen as an optically anisotropic wave plate possessing variable phase retardance *ψ*_PP_, which is made up of a transparent glass cell filled with LC molecules (LCC1413-A, Thorlabs). The slow axis of this sample is parallel to its plate surface. The two parallel inner faces of the cell wall are coated with transparent conductive films, so that a desired external voltage can be applied across the cell. Remarkably, once applying an AC voltage, the LC molecules reorient themselves from the default arrangement depending to the applied voltage, thereby precisely controlling *ψ*_PP_. As shown in Figure 2(c), under room temperature at 633 nm wavelength incidence, we obtain *ψ*_PP_ = 2*π* at voltage *u* = 0.98 V and *ψ*_PP_ = *π* at *u* = 1.45 V in experiments correspondingly, and the maximum retardance here is about 2.4*π*. At 445 nm wavelength incidence, the maximum retardance is about 4.3*π*, and we have *ψ*_PP_ = 2*π* at *u* = 1.42 V and *ψ*_PP_ = *π* at *u* = 2.05 V. More of the retardance in a wider voltage ranges is shown in Figure S7 of the Supplementary 1, but we focus on the retardance change between *π* and 2*π* in this approach. By applying a fixed voltage, this retardance possesses good temperature stability. Meanwhile, the high-transmission of the LC PP sample is also measured in Figure 2(d), which ensures the good performance of the operation platform. Note that the switching time from the higher voltage to the lower voltage (fall time) in this platform is far less than 1 ms, which enables the quick switching and operation (see Supplementary Material for details).

### 3.2 Experimental realization of 1D switchable differential operations

**Figure 3:**
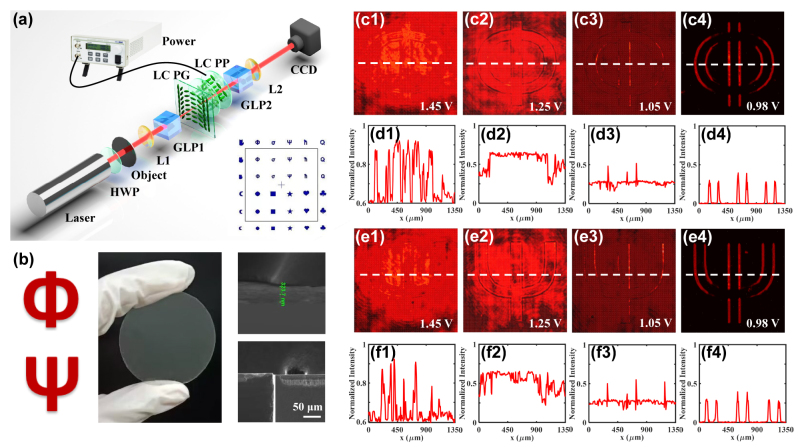
Experimental realization of switchable spatial differential operation for obtaining one-dimensional (1D) edge-enhanced phase images in desired contrasts. (a) Experimental setup. The wavelength of incidence is *λ* = 633 nm. The phase object is placed on the front focal plane of L1, and the LC PG and CCD are placed on the front and back focal planes of L2, respectively. The LC PP adjusts the phase delay of system through an applied voltage. The remaining parameters are the same to that in Figure 1. (b) The photograph of the object and its SEM images. (c1)–(c4) and (e1)–(e4) are the switching results of the edge-enhanced images for two letter objects. The voltages are applied to *u* = 1.45 V, 1.25 V, 1.05 V, and 0.98 V. (d1)–(d4) and (f1)–(f4) are the corresponding normalized intensities across the white dashed lines.

To verify the prediction of the proposed platform in realizing switchable spatial differential operation, we experimentally demonstrate the edge-enhancement procedure for 1D phase imaging. The experimental setup is shown in Figure 3(a) similar to the one in Figure 2(a), but a pure phase object is set at the front focal plane of L1. The distances between the object and L1, L1 and LC PG, LC PG and L2, L2 and CCD are the same to 175 mm. Figure 3(b) show the photograph and SEM image of the phase object, which is manufactured on a 500 μm-thickness glass substrate with different phase patterns by the photo-lithography method, and we select two different letters for the object samples. The optical axis of GLP1 is aligned in the *x*-direction to prepare the initial polarization state. After the light is passed through the LCs, we use GLP2 to release the photons along the *y*-direction and then capture the output intensity by the CCD.

Under the illumination of 633 nm light source, we rotate the optical axis of GLP1 to be aligned in the *x*-direction, and set that of GLP2 in the *y*-direction to release photons. The experimental results show that the bright-field images are obtained when *u* = 1.45 V due to *ψ*_PP_ = *π* (*K*_2_ = 0) (see Figure 3(c1) and (e1)). With the decrease of applied voltage, the edge contrast of the output image is gradually enhanced (see Figure 3(c2), (c3), (e2), and (e3)). When the voltage is adjusted to *u* = 0.98 V with *ψ*_PP_ = 2*π* (*K*_1_ = 0), the distinct differential images along the *x*-direction are acquired (see Figure 3(c4) and (e4)). To indicate the edge-enhancement procedure in more detail, we further plot the normalized intensities across the white dashed lines in these images, as shown in Figure 3(d1)–(d4) and (f1)–(f4). Compared with the case without applying a voltage, with improved contrasts, the edge contrast is gradually improved by dynamically modulating *ψ*_PP_ until obtaining a clear outline. These phenomena satisfy well with the theoretical predictions described in Eq. (3). As an analog, the corresponding 1D dark-field differential operation along the *y*-direction can also be achieved according to Eq. (4). These results demonstrate the capability of the proposed platform in reliable spatial differential operation as well as the potential to perform switchable differential microimaging (see the Supplementary Information for details).

### 3.3 Experimental realization of 2D controllable dark-field differential microscopy

**Figure 4:**
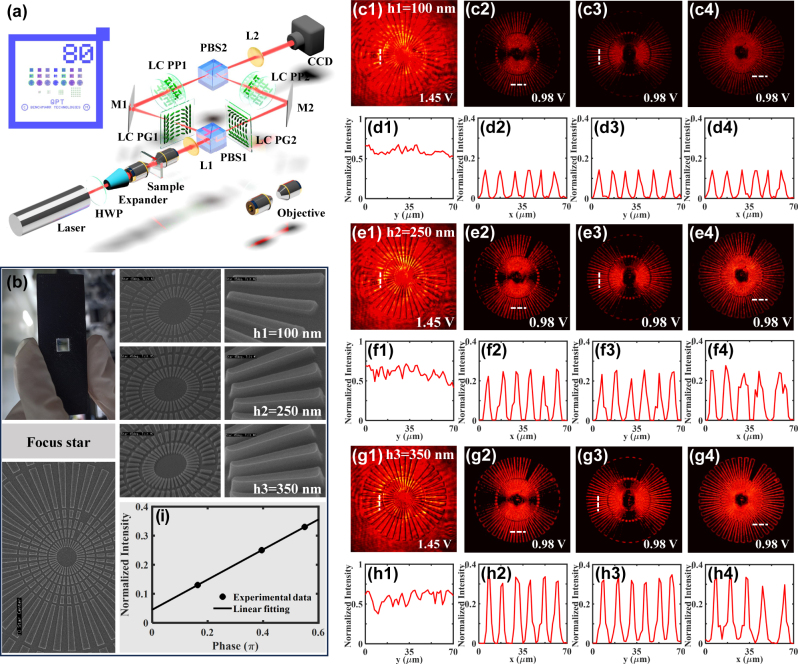
Experimental realization of switchable differential microscopy for obtaining 2D edge-enhanced quantitative phase microscopy targets in desired contrasts. (a) Experimental setup. PBS, polarizing beam splitter. M, mirror. Two different sets of LCs (one of LC PG1 and LC PP1, and the other of LC PG2 and LC PP2) are placed on the confocal plane of L1 and L2 to realize the differential operation along the *x*- and *y*-directions, respectively. The microscopy target (sample) is placed on the front focal plane of L1. A laser expander and a pair of optical microscopes (objective, 0.25 NA) are induced to satisfy the detection in micro-scale. The other parameters are the same to Figure 3. (b) The photograph of the sample and its SEM images with three heights of *h*1 = 100 nm, *h*2 = 250 nm, and *h*3 = 350 *nm*. (c)–(h) Experimental results. Here, (c1)–(c4), (e1)–(e4), and (g1)–(g4) are the switching results of the edge-enhanced images in heights *h*1 = 100 nm, *h*2 = 250 nm, and *h*3 = 350 nm, respectively. (d1)–(d4), (f1)–(f4), and (h1)–(h4) are the corresponding normalized intensities across the white dashed lines. The first column shows the results of bright-field images with applied voltage *u* = 1.45 V. The second and the third columns are the differential results along the *x*-direction (path 1) and the *y*-direction (path 2), respectively. The last column is the results of outputted 2D differential images with applied voltage *u* = 0.98 V. (i) The relationship between the normalized intensity along the image edge and the phase change formed by the three different-height targets.

The proposed scheme can be expanded to the differential microscopy for edge-enhanced 2D micro-imaging. The experimental setup is shown in Figure 4(a). Here, a pair of conventional optical microscopes with objectives (Plan 10×, 0.25 NA, SAGA) as well as a beam expander (GCO-2501, DHC) are introduced to meet the micro-scale detection. A quantitative phase microscopy target (fabricated by Benchmark Technologies, USA) is sandwiched by the objectives and placed on the front focal plane of L1. As shown in Figure 4(b), this target is manufactured on a 1.1 mm-thick Corning Eagle XG glass (refractive index 1.52) by advanced ebeam mask writer and etch. The pure-phase patterns on it are raised by different feature heights. In this scheme, we select the 100 nm-height, 250 nm-height, and 350 nm-height focus stars as the targets. The input light intensity of the experiment is modulated to the same appropriate level to ensure a more impartial comparison and analysis to the detection results of three different-height targets, and also to prevent the damage to CCD camera from excessive exposures.

The optical paths 1 and 2 are designed to obtain the differential results along the *x*- and *y*-directions simultaneously. The first polarizing beam splitter (PBS1) splits the photons into its *H* and *V* polarization components. When the two components are passed through the path 1 and path 2, respectively, the photons after the computing LCs of each path manifest itself as a mixed signal, including the bright-field image and the differential image. The mirrors M1 and M2 reflect the information to the two mutually orthogonal input ports of PBS2. Subsequently, we adopt the PBS2 to release the photons in the *y*-direction (as demonstrated in Eq. (3)) and the *x*-direction (as demonstrated in Eq. (4)) simultaneously. For the 100 nm-height target, the experimental results show that the bright-field images are captured when applying 1.45 V-voltage (*ψ*_PP_ = *π*, *K*_2_ = 0) (see Figure 4(c1) and (d1)). Setting 0.98 V-voltage (*ψ*_PP_ = 2*π*, *K*_1_ = 0), we then obtain the differential images along the *x*-direction (see Figure 4(c2) and (d2)) and the *y*-direction (see Figure 4(c3) and (d3)). According to Eq. (7), by superimposing the output photons of the two paths into one, we finally obtain the 2D differential images, as shown in Figure 4(c4) and (d4). Likewise, we experimentally realize the detection of the 250 nm-height and 350 nm-height targets, as shown in Figure 4(e1)–(h4). It is demonstrated that the 2D edges of the targets can all be clearly extracted, and the captured intensities (i.e., edges’ brightness) show an enhancement with the increase of the height.

Note that the structures of the targets given as 100 nm, 250 nm, and 350 nm are substantial phase changes. And this change can be calculated by $\varphi =\left(2\pi /\lambda \right)nh$ , where *n* denotes the refractive-index change from the target to air, and *h* is the height of the target. For the 633 nm-wavelength incidence, the corresponding phase is about 0.158*π*, 0.395*π*, and 0.55*π*, and we further plot Figure 4(i) to present the detected relationship between the normalized intensity and the phase change with a linear fitting. From the optical differential perspective, the edge of the light intensity enhances as the phase gradient increases, indicating that the 2D edges of the targets with different phase changes can all be clearly visible by our approach. And based on the capability in extracting phase gradient, some methods are applicable to perform direct phase measurements and thickness estimations, such as weak-value modulation [19], bias retardation [43], etc.

### 3.4 Experimental realization of 2D multi-wavelength microscopy imaging

**Figure 5:**
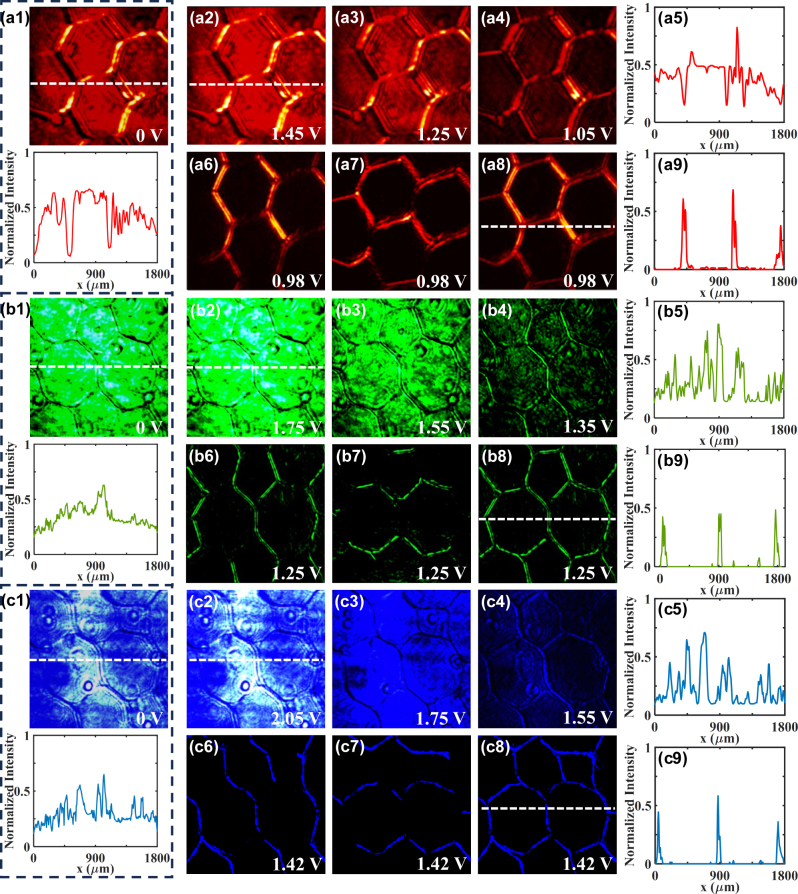
Experimental realization of switchable multi-wavelength differential microscopy for measuring dicotyledon cells in desired contrasts. (a), (b), and (c) are the experimental results under the illuminations of 633 nm-wavelength (red light), 532 nm-wavelength (green light), and 445 nm-wavelength (blue light). Here, (a1), (b1), and (c1) are the sample images with no applied voltage. (a2)–(a4) show the edge-enhanced images with *u* = 1.45 V, 1.25 V, and 1.05 V, respectively. (a6)–(a8) show the differential results with *u* = 0.98 V, containing the 1D edge images along the *x*-direction and the *y*-direction, as well as their complete 2D edge image. (a5) and (a9) are the normalized intensities across (a2) and (a8). (b2)–(b4) show the edge-enhanced images with *u* = 1.75 V, 1.55 V, and 1.35 V, respectively. (b6)–(b8) are the differential results with *u* = 1.25 V, containing the 1D edge images along the *x*-direction and the *y*-direction, as well as their complete 2D edge image. (b5) and (b9) are the normalized intensities across (b2) and (b8). (c2)–(c4) show the edge-enhanced images with *u* = 2.05 V, 1.75 V, and 1.55 V, respectively. (c6)–(c8) are the differential results with *u* = 1.42 V, containing the 1D edge images along the *x*-direction and the *y*-direction, as well as their complete 2D edge image. (c5) and (c9) are the normalized intensities across (c2) and (c8).

We finally utilize our scheme to the 2D microscopy imaging application for biological cells. We use a three-wavelength mode laser (LSR-RGB-500, LASE EVER) to provide light source in this part. By replacing the quantitative phase microscopy target in Figure 4(a) with transparent dicotyledonous plant slices, we obtain the bioimaging results under illumination of 633 nm-wavelength incidence (red light) (see Figure 5(a1)–(a9)). Figure 5(a1) is the image of dicotyledonous plant cell with no applied voltage. Figure 5(a2) is the 2D bright-field image of the cell under 1.45 V-voltage. The two images both show low edge contrast and hard to distinguish the cell feature, which can be attributed to the pure phase nature of the specimens. To observe the more detailed cell, we adjust the external voltage of the proposed platform from *u* = 1.45 V (*ψ*_PP_ = *π*, *K*_2_ = 0) to *u* = 0.98 V (*ψ*_PP_ = 2*π*, *K*_1_ = 0). In this process, the edge-enhanced images of the cell are switched in Figure 5(a3) and (a4). The differential images along the *x*-direction, *y*-direction, as well as their 2D results, are finally captured in Figure 5(a6)–(a8). It is demonstrated that with the modulation of the applied voltage, the outlines of images gradually become clearer, and the sharp 2D edges of the cell are acquired with improved contrast. The normalized intensity cross sections of Figure 5(a2) and (a8) are described by Figure 5(a5) and (a9) correspondingly. These results reveal the flexible regulatory of our approach for differential microscopy and bioimaging.

To testify the multi-wavelength capability of our scheme, we further perform the experiment under the illumination of 532 nm-wavelength incidence (green light) and 445 nm-wavelength incidence (blue light). The results are shown in Figure 5(b1)–(c9), respectively. Figure 5(b1) and (c1) are the sample image with no applied voltage, showing blurred image contours. By applying the voltages, we obtain the bright-field image of the cell at *u* = 1.75 V and *u* = 2.05 V (*ψ*_PP_ = *π*, *K*_2_ = 0) with low-contrast edges, and their normalized intensities are further captured in Figure 5(b5) and (c5). By adopting our operation platform with modulated applied voltages, the edge-enhanced images can be switched with gradually improved contrasts (see Figure 5(b3), (b4), (c3), and (c4)). The strict differential results along the *x*- and *y*-directions as well as their 2D results are then acquired as image edges at *u* = 1.25 V and *u* = 1.42 V (*ψ*_PP_ = 2*π*, *K*_1_ = 0) with regular normalized intensity (see Figure 5(b6)–(b9) and (c6)–(c9)). It is demonstrated that the 2D bioimaging can be realized under multi-wavelength conditions by our approach.

From a theoretical point of view, the imaging capability under multiple wavelengths of our approach can be mainly attributed to the birefringence working mechanism and geometric phase of the computing LCs. The LC PG possesses a phase retardation *π* at working wavelength of 633 nm, so that photons after this plate will reverse their handedness absolutely and form an additional Pancharatnam–Berry phase gradient (i.e., the beam shift, Δ*x* in the real-space) to enable optical operation and imaging. Although at some working wavelengths such as 445 nm, a few photons after passing through the LC PG may still maintain their initial handedness, they subsequently recombine to the initial polarization and are eliminated by the analyzer. Therefore, only the critical edge information is ultimately extracted, and we can achieve the image edge-enhancement as well as clear microscopy imaging under multi-wavelength conditions. Compared with the conventional differential-interference-contrast microscopy and optical phase-contrast microscopy, the proposed differential microscopy can not only realize the 2D differential operations of pure phase objects and color images, but also quick-switch the edge-enhanced images in desired contrasts with flexible operations. Moreover, from the perspective of rigorous phase-contrast imaging applications, such as detecting the biological motion under low-light levels, the proposed scheme is expected to serve as an important tool to obtain high signal-to-noise ratio results with the illumination of quantum-correlated sources [47].

## 4 Conclusions

In conclusion, we have proposed and experimentally realized the electrically switched differential microscopy with the aid of the computing liquid-crystal platform. In our scheme, the LC PG with PB phase gradient separates the input phase object into two replicas of opposite circular polarization components, promoting the 2D image edge detection with optical differential operations. The electrically switched LC PP provides an additional phase retardance for system with about 310 μs-switching-time, so that the output mode forming edge-enhanced images can be flexibly tuned in desired contrasts. Applying the proposed platform to the differential microscopy for practical imaging applications, the 2D differential results of three different-height microscopy targets are experimentally extracted. Meanwhile, significant signals at the edges of transparent biological cells are detected with improved contrasts than the initial images, which overcome the invisibility of the pure phase objects due to the transparent nature. If utilized to processing amplitude objects, this approach can achieve the dynamic switching of the bright-field and the dark-field images via flexible regulation. Besides, based on the capability in extracting phase gradient contained in the image edge information, direct phase measurements and thickness estimations may be performed with additional optical operations. Since the computing LC possesses the unique geometric phase features not limited to application under one single frequency, our scheme also enables the 2D edge detection of transparent micro-structures under multiple wavelengths. These results provide the pathway of forming multifunctional and miniaturized differential microscopy, and may form a developing practical technology with considerable application prospects toward all-optical image processing, machine vision and deep learning [48], [49], and real-time medical diagnosis as well as air quality testing [50].

## Supplementary Material

Supplementary Material Details
