# Supplementary material for: Electrically-switched differential microscopy based on computing liquid-crystal platforms
Source: Nanophotonics. 2024 Jan 24;13(3):327–38. doi: 10.1515/nanoph-2023-0688 (PMC11502053; doi:10.1515/nanoph-2023-0688)
Supplement: Supplementary file 1 — Supplementary Material Details [file j_nanoph-2023-0688_suppl_001.docx]

Supplement 1

Shuoqing Liu, Dandan Zheng, Qiang Yang, Shizhen Chen^[[1]](#footnote-1)^, Shuangchun Wen, and Hailu Luo

Supplementary Information: Electrically-switched differential microscopy based on computing liquid-crystal platforms

This document provides supplementary information to "Electrically-switched differential microscopy based on computing liquid-crystal platforms".

1. **General optical differential operation with the computing liquid crystal**

The designed platform relies mainly on the liquid crystal (LC) polarization grating (PG) and the electrically-switched LC phase plate (PP). We here demonstrate the general optical differential operation with the computing LC. The proposed LC PG is a typical diffractive optical element, with a constant phase retardance $\psi=\pi$ at 633 nm wavelength and an efficient Pancharatnam-Berry phase (PB phase) endowed by its artificially designed structure. The LC molecules with anisotropic shapes are often self-assemble with a preferred direction of orientation to form a spatial-variant arrangement (i.e., local optical axis) in period $d=10 \mathrm{mm}$, and the general position-dependent Jones matrix of this sample can be described as [1]

$T_{\mathrm{PG}}\approx i\sin\left( \psi/2 \right)\left[ \begin{matrix} cos2\rho& sin2\rho\\ sin2\rho& -cos2\rho\end{matrix} \right]$.  (1)

Here, $\rho\left( x,y \right)={\pi x}/d$ [or $\rho\left( x,y \right)={\pi y}/d$] represents the orientation of sample’s local optical axis. Suppose that the incidence of left-handed circular polarized (LCP) beam is passed through this PG (located at the Fourier plane), the output field can be given by

$$E_{\mathrm{out}}=T_{\mathrm{PG}}\cdot\mathbf{e}_{+}\simeq\left[ \begin{matrix} cos2\rho& sin2\rho\\ sin2\rho& -cos2\rho\end{matrix} \right]\cdot\left[ \begin{matrix} 1 \\ +i \end{matrix} \right]$$

$=e^{+i2\rho}\cdot\left[ \begin{matrix} 1 \\ -i \end{matrix} \right]=e^{+i2\rho}\cdot\mathbf{e}_{-}$. (2)

Here, $\mathbf{e}_{+}$ and $\mathbf{e}_{-}$ denote the electric-field vectors of LCP and right-handed circular polarized (RCP) components, respectively. It is found that the incident photons reverse their chirality from the LCP beam to the RCP beam, and an additional real-space PB phase $\varphi_{\mathrm{PB}}\left( x,y \right)=e^{+i2\rho\left( x,y \right)}$ is induced in this process. This PB phase gradient manifests itself as the spin-dependent shift in the momentum space $\Delta k\left( x,y \right)=\nabla\varphi_{\mathrm{PB}}\left( x,y \right)$. If converted into the real-space by Fourier transformations after propagation a distance $z$, a real-space beam shift $\Delta x={\Delta kz}/{k_{0}}$ (or $\Delta y={\Delta kz}/{k_{0}}$) is formed inversely proportional to the wavenumber $k_{0}$ [2]. Similarly, when the RCP beam is passed through the LC PG, it will convert into the LCP one with an additional PB phase $\varphi_{\mathrm{PB}}\left( x,y \right)=e^{-i2\rho\left( x,y \right)}$, and ultimately causes a real-space beam shift in the opposite direction. The unique capability of LC PG in manipulating the polarization and phase of photons as well as the induction of spin-dependent shift, make it an excellent candidate in optical differential operation.

A linearly polarized (LP) beam can be regarded as a superposition of the LCP and the RCP beams with the same amplitude and opposite spin angular momentums. Consider the input LP beam selected by the polarizer with the optical axes in the *x*-direction, i.e., with horizontal (*H*) polarization, which can be described as

$E_{\mathrm{in}}\left( x,y \right)=E_{\mathrm{in}}\left( x,y \right)\mathbf{e}_{x}\propto E_{\mathrm{in}}\left( x,y \right)\left( \mathbf{e}_{+}+\mathbf{e}_{-} \right)$. (3)

After this *H*-polarized beam is passed through the computing LC PG, the electric field satisfies

$$E_{\mathrm{PG}}\left( x,y \right)=E_{\mathrm{in}}\left( x+\Delta x,y \right)\left[ \begin{matrix} 1 \\ i \end{matrix} \right]+E_{\mathrm{in}}\left( x-\Delta x,y \right)\left[ \begin{matrix} 1 \\ -i \end{matrix} \right]=\left[ E_{\mathrm{in}}\left( x+\Delta x,y \right)+E_{\mathrm{in}}\left( x-\Delta x,y \right) \right]\mathbf{e}_{x}+i\left[ E_{\mathrm{in}}\left( x+\Delta x,y \right)-E_{\mathrm{in}}\left( x-\Delta x,y \right) \right]\mathbf{e}_{y}\simeq{2E}_{\mathrm{in}}\left( x,y \right)\mathbf{e}_{x}+i2\Delta x\frac{\partial E_{\mathrm{in}}\left( x,y \right)}{\partial x}\mathbf{e}_{y}.$$

(4)

Here, the last calculation holds up due to the particularly small beam shift $\Delta x$ (much smaller than the field distribution), and a mixture of bright-field signal and dark-field differentiation is formed obviously. If only the photons in the *y*-direction are allowed to pass through, we can acquire the pure differentiation of the input field along the *x*-direction, that is,

$E_{out-y}\left( x,y \right)=i2\Delta x\frac{\partial E_{\mathrm{in}}\left( x,y \right)}{\partial x}\mathbf{e}_{y}\propto\Delta x\frac{\partial E_{\mathrm{in}}\left( x,y \right)}{\partial x}\mathbf{e}_{y}$. (5)

It is worth to mention that the complete handedness conversion only appears at 633 nm wavelength incidence, since the phase retardance of LC is π at this time, satisfying the half-wave condition. Although at some working wavelengths such as 445 nm, a few photons after passing through the LC PG may still maintain their initial handedness, they subsequently recombine to the initial *H*-polarization and are eliminated by the analyzer. Therefore, only the critical edge information is ultimately extracted. And for any incident light with multi-wavelength, we can achieve the image edge-enhancement as well as clear microscopy imaging, as illustrated on Eqs. (4) and (5).

As an analogue, suppose the incidence of *y*-direction, i.e., with vertical (*V*) polarization, which can be described as

$E_{\mathrm{in}}\left( x,y \right)=E_{\mathrm{in}}\left( x,y \right)\mathbf{e}_{y}\propto iE_{\mathrm{in}}\left( x,y \right)\left( \mathbf{e}_{-}-\mathbf{e}_{+} \right)$. (6)

After the light-PG interaction, the electric field satisfies as

$$E_{\mathrm{PG}}\left( x,y \right)=iE_{\mathrm{in}}\left( x,y-\Delta y \right)\left[ \begin{matrix} 1 \\ -i \end{matrix} \right]-iE_{\mathrm{in}}\left( x,y+\Delta y \right)\left[ \begin{matrix} 1 \\ +i \end{matrix} \right]=i\left[ E_{\mathrm{in}}\left( x,y-\Delta y \right)-E_{\mathrm{in}}\left( x,y+\Delta y \right) \right]\mathbf{e}_{\boldsymbol{x}}\boldsymbol{+}\left[ E_{\mathrm{in}}\left( x,y-\Delta y \right)+E_{\mathrm{in}}\left( x,y+\Delta y \right) \right]\mathbf{e}_{y}\simeq i2\Delta y\frac{\partial E_{\mathrm{in}}\left( x,y \right)}{\partial y}\mathbf{e}_{x}+2E_{\mathrm{in}}\left( x,y \right)\mathbf{e}_{y}$$

(7)

with a tiny beam shift $\Delta y$. Therefore, we can ultimately obtain the differentiation along the *y*-direction by releasing the photons in the *x*-direction

$E_{out-x}\left( x,y \right)=i2\Delta y\frac{\partial E_{\mathrm{in}}\left( x,y \right)}{\partial y}\mathbf{e}_{x}\propto\Delta y\frac{\partial E_{\mathrm{in}}\left( x,y \right)}{\partial y}\mathbf{e}_{x}$. (8)

From Eqs. (5) and (8), the one-dimensional (1D) differential results in the *x*- or *y*-directions are presented, respectively.

1. **Electrically-switched 1D optical differential operation based on the computing LCs**

To form a computing LC platform aiming at high-performance information processing, we specifically consider an electronically-switched LC PP after the LC PG in the system, as shown in Fig. S1(a). The He-Ne laser generates the input Gaussian beam, and the half-wave plate (HWP) adjusts its intensity to prevent damage to the charge-coupled device (CCD). Lenses L1 and L2 ($f_{1}=f_{2}=175 \mathrm{mm}$) form a 4f system. Among them, the polarization axes of the two Glan polarizers (GLP1 and GLP2) are orthogonal to each other, with the LC PG on the confocal plane, followed by the LC PP. The object is placed on the front focal plane of L1 (a pair of optical microscopes should be introduced for imaging of transparent specimens in the micro-scale). The matrix of the LC PP is given by [1]

$J\left( \psi_{PP} \right)=\left[ \begin{matrix} e^{-i{\psi_{PP}}/2} & 0 \\ 0 & e^{+i{\psi_{PP}}/2} \end{matrix} \right]$. (9)

Here, $\psi_{PP}$ represents the phase retardance inherent to the plate, which also provides a phase delay for the operation system through precise modulation of applied voltages. Applying the coordinate rotation matrix

$R\left( \rho_{PP} \right)=\left[ \begin{matrix} \cos\rho_{PP} & \sin\rho_{PP} \\ -\sin\rho_{PP} & \cos\rho_{PP} \end{matrix} \right]$ (10)

with $\rho_{PP}$ indicating the local axes of the LC PP, the position-dependent transmission matrix of the plate can be described as

$$T_{\mathrm{PP}}=R\left( -\rho_{\mathrm{PP}} \right)J\left( \psi_{\mathrm{PP}} \right)R\left( \rho_{\mathrm{PP}} \right)=\left[ \begin{matrix} \cos\frac{\psi_{\mathrm{PP}}}{2}-i\sin\frac{\psi_{\mathrm{PP}}}{2}\cos\left( 2\rho_{\mathrm{PP}} \right) & -i\sin\frac{\psi_{\mathrm{PP}}}{2}\sin\left( 2\rho_{\mathrm{PP}} \right) \\ -i\sin\frac{\psi_{\mathrm{PP}}}{2}\sin\left( 2\rho_{\mathrm{PP}} \right) & \cos\frac{\psi_{\mathrm{PP}}}{2}+i\sin\frac{\psi_{\mathrm{PP}}}{2}\cos\left( 2\rho_{\mathrm{PP}} \right) \end{matrix} \right].$$

(11)

In our scheme, setting $\rho_{\mathrm{PP}}=\pi/4$, Eq. (11) can be simplified as

$T_{\mathrm{PP}}=\left[ \begin{matrix} \cos\frac{\psi_{\mathrm{PP}}}{2} & -i\sin\frac{\psi_{\mathrm{PP}}}{2} \\ -i\sin\frac{\psi_{\mathrm{PP}}}{2} & \cos\frac{\psi_{\mathrm{PP}}}{2} \end{matrix} \right]$. (12)

Combined with Eq. (4) under the incidence of *H*-polarized beam, the electric field after the computing LCs (composed of the LC PG and the LC PP) is given by

$$E_{out-1}\left( x,y \right)\simeq\left[ \begin{matrix} \cos\frac{\psi_{\mathrm{PP}}}{2} & -i\sin\frac{\psi_{\mathrm{PP}}}{2} \\ -i\sin\frac{\psi_{\mathrm{PP}}}{2} & \cos\frac{\psi_{\mathrm{PP}}}{2} \end{matrix} \right]\left[ \begin{matrix} 2E_{\mathrm{in}}\left( x,y \right) \\ i2\Delta x\frac{\partial E_{\mathrm{in}}\left( x,y \right)}{\partial x} \end{matrix} \right]=\left[ 2\cos\frac{\psi_{\mathrm{PP}}}{2}E_{\mathrm{in}}\left( x,y \right)+2\Delta x\sin\frac{\psi_{\mathrm{PP}}}{2}\frac{\partial E_{\mathrm{in}}\left( x,y \right)}{\partial x} \right]\mathbf{e}_{x}+\left[ -i2\sin\frac{\psi_{\mathrm{PP}}}{2}E_{\mathrm{in}}\left( x,y \right)+i2\Delta x\cos\frac{\psi_{\mathrm{PP}}}{2}\frac{\partial E_{\mathrm{in}}\left( x,y \right)}{\partial x} \right]\mathbf{e}_{y}.$$

(13)

From the perspective of differential operation, only the *y*-direction here is allowed to pass through [see Fig. S1(b)], and the field evolves as

$E_{out-1}\left( x,y \right)=\left[ -i2\sin\frac{\psi_{\mathrm{PP}}}{2}E_{\mathrm{in}}\left( x,y \right)+i2\Delta x\cos\frac{\psi_{\mathrm{PP}}}{2}\frac{\partial E_{\mathrm{in}}\left( x,y \right)}{\partial x} \right]\mathbf{e}_{y}$. (14)

Combined with Eq. (7) under the incidence of *V*-polarized beam, the electric field after the computing LCs is given by

$$E_{out-2}\left( x,y \right)\simeq\left[ \begin{matrix} \cos\frac{\psi_{\mathrm{PP}}}{2} & -i\sin\frac{\psi_{\mathrm{PP}}}{2} \\ -i\sin\frac{\psi_{\mathrm{PP}}}{2} & \cos\frac{\psi_{\mathrm{PP}}}{2} \end{matrix} \right]\left[ \begin{matrix} i2\Delta y\frac{\partial E_{\mathrm{in}}\left( x,y \right)}{\partial y} \\ 2E_{\mathrm{in}}\left( x,y \right) \end{matrix} \right]=\left[ i2\Delta y\cos\frac{\psi_{\mathrm{PP}}}{2}\frac{\partial E_{\mathrm{in}}\left( x,y \right)}{\partial y}-i2sin\frac{\psi_{\mathrm{PP}}}{2}E_{\mathrm{in}}\left( x,y \right) \right]\mathbf{e}_{x}+\left[ 2\Delta y\sin\frac{\psi_{\mathrm{PP}}}{2}\frac{\partial E_{\mathrm{in}}\left( x,y \right)}{\partial y}+2cos\frac{\psi_{\mathrm{PP}}}{2}E_{\mathrm{in}}\left( x,y \right) \right]\mathbf{e}_{y}.$$

(15)

In this case, only the *x*-direction is allowed to pass through [see Fig. S1(b)], and then

$E_{out-2}\left( x,y \right)=\left[ i2\Delta y\cos\frac{\psi_{\mathrm{PP}}}{2}\frac{\partial E_{\mathrm{in}}\left( x,y \right)}{\partial y}-i2sin\frac{\psi_{\mathrm{PP}}}{2}E_{\mathrm{in}}\left( x,y \right) \right]\mathbf{e}_{x}$. (16)

According to Eqs. (S14) and (S16), the output fields of the two operation paths are all mixtures containing the bright-field and differentiation information, and the operation results ultimately obtained mainly rely on the phase retardance $\psi_{\mathrm{PP}}$. Since the bright-field results and the differential results are closely related to the function $\sin\left( {\psi_{\mathrm{PP}}}/2 \right)$ and $\cos\left( {\psi_{\mathrm{PP}}}/2 \right)$ correspondingly, the flexible control of the output results can be achieved in modes of bright-field image, edge-enhanced image, and the high-contrast edge image by applying the voltage to modulate the desired $\psi_{\mathrm{PP}}$.

**Fig. S1**. Electrically-switched 1D optical differentiator based on computing LCs. (a) Experimental setup. Laser, He-Ne Laser (Thorlabs). HWP, half-wave plate. Object, pure phase object. L, lens (focal length 175 mm). GLP, Glan polarizer. PG, liquid-crystal polarization grating. PP, liquid-crystal phase plate. Power, power supply. CCD, charge-coupled device. Objective, microscopes. (b) Optical differential operation along the *x*-direction and the *y*-direction, respectively. The bright-field image and edge image can be electrically-switched by phase retardances $\psi_{\mathrm{PP}}=\pi$ and $\psi_{\mathrm{PP}}=2\pi$.

The 1D optical differential results of the proposed approach are shown in Fig. S2. This result is obtained by experiments under room temperature at 633 nm wavelength incidence. We take a pure amplitude object [Fig. S2(a1)] and a low-contrast phase object [Fig. S2(b1)] as the samples. Figures S2(a2) and S2(b2) are the result without an applied voltage, showing undefined edges. When the voltage is applied to 1.45 V (i.e., $\psi_{PP}=\pi$), we acquire the bright-field image with hazy outlines [Figs. S2(a3) and S2(b3)]. Figures S2(a4, a5) and S2(b4, b5) are the edge-enhancement process with voltage variation. When a voltage of 0.98 V (i.e., $\psi_{PP}=2\pi$) is applied, the excess signal of input light is filtered, and only distinct edges of the image are retained, corresponding to the pure amplitude or phase gradient information [Figs. S2(a6) and S2(b6)]. Compared with the initial images, the edge contrast is gradually improved. Figures S2(c1-c5) and S2(d1-d5) are the 3D stereo results corresponding to Figs. S2(a2-a6) and S2(b2-b6). These results further demonstrate the capability of the optical differentiation to extract the edge of the originally blurred objects. Therefore, by applying the voltage to modulate the phase retardance $\psi_{PP}$ of system, we can achieve the 1D contour visualization of transparent or blurred objects flexibly.

**Fig. S2**. Experimental results of the electrically-switched 1D optical differentiator. (a1) and (b1) are the amplitude object and the phase object. (a2) and (b2) are the image processing results of no applied voltage. (a3-a6) and (b3-b6) are the edge-enhancement results under voltages of 1.45 V, 1.25 V, 1.05 V, and 0.98 V. (c1-c5) and (d1-d5) are the 3D stereo results corresponding to Figs. S2(a2-a6) and S2(b2-b6). Scale bar: 150 μm.

1. **Electrically-switched two-dimensional differential microscopy based on the computing LCs**

To realize the electrically-switched two-dimensional (2D) optical analog computing, two optical paths are applied together to form a complete computing LC platform with the help of two mutually orthogonal polarizing beam splitters (PBS), as shown in Fig. 4(a) of the main text. Based on the discussions of Eqs. (14) and (16), the final output field through the whole platform is given by

$$E_{\mathrm{out}}\left( x,y \right)=E_{out-1}\left( x,y \right)+E_{out-2}\left( x,y \right)=\left[ -i2\sin\frac{\psi_{\mathrm{PP}}}{2}E_{\mathrm{in}}\left( x,y \right)+i2\Delta x\cos\frac{\psi_{\mathrm{PP}}}{2}\frac{\partial E_{\mathrm{in}}\left( x,y \right)}{\partial x} \right]\mathbf{e}_{y}+\left[ i2\Delta y\cos\frac{\psi_{\mathrm{PP}}}{2}\frac{\partial E_{\mathrm{in}}\left( x,y \right)}{\partial y}-i2sin\frac{\psi_{\mathrm{PP}}}{2}E_{\mathrm{in}}\left( x,y \right) \right]\mathbf{e}_{x}=-i2\sin\frac{\psi_{\mathrm{PP}}}{2}\left[ E_{\mathrm{in}}\left( x,y \right)\mathbf{e}_{y}+E_{\mathrm{in}}\left( x,y \right)\mathbf{e}_{x} \right]+i2cos\frac{\psi_{\mathrm{PP}}}{2}\left[ \Delta x\frac{\partial E_{\mathrm{in}}\left( x,y \right)}{\partial x}\mathbf{e}_{y}+\Delta y\frac{\partial E_{\mathrm{in}}\left( x,y \right)}{\partial y}\mathbf{e}_{x} \right].$$

(17)

Obviously, when phase retardance $\psi_{\mathrm{PP}}=\pi$, the field evolves as

$E_{\mathrm{out}}\left( x,y \right)\propto E_{\mathrm{in}}\left( x,y \right)\mathbf{e}_{y}+E_{\mathrm{in}}\left( x,y \right)\mathbf{e}_{x}$ (18)

corresponding to the 2D output image in bright-field. With the increase of $\psi_{\mathrm{PP}}$, the mixture consists of the bright-field part with decreasing proportion and the differentiation part with increasing proportion, so that we can acquire the edge-enhanced image with improved contrasts. When $\psi_{\mathrm{PP}}=2\pi$, the field evolves as

$E_{\mathrm{out}}\left( x,y \right)\propto\Delta x\frac{\partial E_{\mathrm{in}}\left( x,y \right)}{\partial x}\mathbf{e}_{y}+\Delta y\frac{\partial E_{\mathrm{in}}\left( x,y \right)}{\partial y}\mathbf{e}_{x}$. (19)

Under this circumstance, we can obtain the output image in a superposition of the pure image edge along the *x-* and *y-*directions (i.e., the 2D image edge detection), possessing extremely improved high contrasts. The imaging results are shown in Fig. S3. We select three patterns from the amplitude object as samples. With the increase of voltage, the edge contrast of output images is improved. The pure 2D edge is obtained at $\psi_{\mathrm{PP}}=2\pi$, and the switch from 2D bright-field to dark-field differentiation is realized.

Remarkably, for pure phase-contrast objects with phase distribution $\varphi\left( x,y \right)$ (for example the transparent biological cells and tissues), we have

$E_{\mathrm{in}}\left( x,y \right)=e^{i\varphi\left( x,y \right)}$. (20)

Then, the output image in normal optical microscopes only experiences a phase change. Therefore, the output field in Eq. (S17) can be further expressed as

$$E_{\mathrm{out}}\left( x,y \right)=-i2\sin\frac{\psi_{\mathrm{PP}}}{2}\left[ e^{i\varphi\left( x,y \right)}\mathbf{e}_{y}+e^{i\varphi\left( x,y \right)}\mathbf{e}_{x} \right]+i2cos\frac{\psi_{\mathrm{PP}}}{2}e^{i\varphi\left( x,y \right)}\left[ \Delta x\frac{\partial\varphi\left( x,y \right)}{\partial x}\mathbf{e}_{y}+\Delta y\frac{\partial\varphi\left( x,y \right)}{\partial y}\mathbf{e}_{x} \right].$$

(21)

As a result, the output field at $\psi_{\mathrm{PP}}=\pi$ is given by

$E_{\mathrm{out}}\left( x,y \right)\propto e^{i\varphi\left( x,y \right)}\mathbf{e}_{x}+e^{i\varphi\left( x,y \right)}\mathbf{e}_{y}$ (22)

corresponding to the 2D result of bright-field image. Distinctively, the output field at $\psi_{\mathrm{PP}}=2\pi$ evolves as

$E_{\mathrm{out}}\left( x,y \right)\propto e^{i\varphi\left( x,y \right)}\left[ \Delta x\frac{\partial\varphi\left( x,y \right)}{\partial x}\mathbf{e}_{y}+\Delta y\frac{\partial\varphi\left( x,y \right)}{\partial y}\mathbf{e}_{x} \right]$. (23)

From the perspective of experimental detection, the output light intensity captured by the CCD is proportional to the square of the mode value of the electric field

$I_{\mathrm{out}}\left( x,y \right)\propto\left| E_{\mathrm{out}}\left( x,y \right) \right|^{2}$. (24)

And then the intensity corresponding to Eq. (23) is detected by

$I_{\mathrm{out}}\left( x,y \right)\propto\left| \Delta x\frac{\partial\varphi\left( x,y \right)}{\partial x} \right|^{2}+\left| \Delta y\frac{\partial\varphi\left( x,y \right)}{\partial y} \right|^{2}$ (25)

since $\left| e^{i\varphi\left( x,y \right)} \right|^{2}\propto1$, showing 2D pure phase gradient of objects. It is demonstrated that the phase gradient manifests itself as the intensity-contrast image (edge image) after the proposed electrically-switched operation platform, which makes it feasible to realize the controllable dark-filed differential microscopy.

**Fig. S3.** Experimental results of the electrically-switched 2D optical differentiator. Panels (a1-c1) are the detection samples with three different patterns under the no voltage case. Panels (a2-c2) and (a3-c3) are the results under applied voltages of 1.45 V and 1.25 V. Panels (a4-c4) and (a5-c5) are the differential result along the *y*-direction and the *x*-direction under applied voltage of $u=0.98V$. Panels (a6-c6) are the 2D dark-field differential results.

Based on the above fundamentals, we can obtain the edge-enhancement of transparent phase object, as shown in Fig. S4. Three microscopic targets with different heights of $100 \mathrm{nm}$, $250 \mathrm{nm}$, and $350 \mathrm{nm}$ are chosen as the samples. It is found that the imaging mode can be manipulated by the applied voltage, and pure image edge can be extracted for a 0.98 V-voltage is applied. Although the light intensity in edge detection process will decrease as the target height decreases, we still obtain clear image edges for all the samples, indicating the good capability of this approach in image processing.

This approach is also applicable to achieving the bioimaging of transparent cells, as shown in Fig. S5. We choose the sliced stem of nymphaea tetragona as sample under illuminations of three wavelengths, i.e., 633 nm (red light), 523 nm (green light), and 445 nm (blue light). Panels (a1-c1) are the initial case without an applied voltage, showing a fuzzy cell contour due to its transparent nature. Panels (a2-c2) show the case with applied voltages of 1.45 V, 1.75 V, and 2.05 V, corresponding to the system phase retardance $\psi_{PP}=\pi$ at the three wavelengths, and the output signals can be regarded as bright-field images at this time. Panels (c1-c3) are the results on the edge-enhancement process. It is found that in the absence of applied voltage and the bright-field conditions, the output images present low contrasts and hard to distinguish the two-dimensional (2D) profile of the cell. By modulating the applied voltage, the outlines of images gradually become clearer, meaning that the contrast is enhanced. Panels (a4-c4) show the sharp 2D edges of the cell, corresponding to the pure phase gradient at $\psi_{PP}=2\pi$. These results further demonstrate the flexible application of the proposed microscopy in cell profile recognition and biological imaging.

**Fig. S4.** Experimental results of the proposed differential microscopy enabled image edge-enhancement. (a1-c1) are the detection target with three heights of $h1=100 nm$, $h2=250 nm$, and $h3=350 nm$. (a1-a5), (b1-b5), and (c1-c5) are the imaging results corresponding to these three targets. The voltages of 0V, 1.45 V, 1.25 V, 1.05 V, and 0.98 V are applied.

**Fig. S5.** Experimental results of the proposed differential microscopy enabled bioimaging for sliced stem of nymphaea tetragona. Light source of three wavelengths 633 nm (red light), 532 nm (green light), and 445 nm (blue light) are utilized. Panels (a1-c1) are the images in the no-voltage case. Figures (a2-a4) correspond to the voltages of 1.45 V, 1.25 V, and 0.98 V. Figures (b2-b4) correspond to the voltages of 1.75 V, 1.55 V, and 1.25 V. Figures (c2-c4) are the imaging results under voltages of 2.05 V, 1.75 V, and 1.42 V.

1. **Features of the computing LCs**
   1. **Transfer function of the computing LCs**

The transfer function $H\left( k_{x},k_{y} \right)$, which determines the spatial transform between the incident and reflected electric fields in the momentum space, is expressed as

$H\left( k_{x},k_{y} \right)=\frac{\tilde{E}_{\text{out}}\left( k_{x},k_{y} \right)}{\tilde{E}_{\text{in}}\left( k_{x},k_{y} \right)}$. (26)

Here, $k_{x}=x/\left( \lambda f \right)$ and $k_{y}=y/\left( \lambda f \right)$ with $\lambda=633 \mathrm{nm}$ and $f=175 \mathrm{mm}$ corresponding to the wavelength of incidence and the focal length of lenses. This transfer function can be regarded as a good indicator to demonstrate the performance of differential operation. The experimental setup to measure $H\left( k_{x},k_{y} \right)$ is shown in Fig. S6(a). As an upgrade to Fig. S1(a), the path 1 and the path 2 are two orthogonal optical paths to realize the differential operation along the *x*- and *y*- directions, respectively. The first polarizing beam splitter (PBS1) splits the input beam into two orthogonal polarized components, which releases the photons in the *x*-direction for path 1 and the photons in the *y*-direction for path 2. The optical axes of LC PG1 and LC PG2 are orthogonal to each other. The PBS2 receives the photons from path 1 and path 2, and then release them in the *y*-direction and the *x*-direction correspondingly, so as to output a complete 2D result of differential operation. Combined with Eq. (24), the angular spectrum $\tilde{E}_{\text{out}}\left( k_{x},k_{y} \right)$ can be obtained by capturing the output intensity $I_{\mathrm{out}}\left( x,y \right)$ and transforming $E_{\mathrm{out}}\left( x,y \right)$ from the position space to the momentum space

$\tilde{E}_{\text{out}}\left( k_{x},k_{y} \right)=\iint E_{\text{out}}\left( x,y \right)\exp\left[ -i\left( k_{x}x+k_{y}y \right) \right]dxdy$. (27)

As shown in Fig. S6(b), by placing the CCD before L1, the incident intensity is captured, and $\tilde{E}_{\text{in}}\left( k_{x},k_{y} \right)$ can be calculated. Placing the CCD after L2, the 2D output intensity can be captured. Specially, if place the CCD after L2 when the photons only pass through the path 1 or path 2, the output intensities containing the differential results along the *x*-direction and the *y*-direction can be recorded, and their angular spectrums $\tilde{E}_{\text{out}}\left( k_{x},k_{y} \right)$ are also calculated. On these bases, to show the operation performance of the proposed platform, the transfer function $H\left( k_{x},k_{y} \right)$ when ${k_{x}}/{k_{0}}=0$ and ${k_{y}}/{k_{0}}=0$ are experimentally extracted in Figs. S6(c) and S6(d). The experimental results satisfy well with the theoretical predictions. Besides, to realize the high-contrast measurement here, the phase retardances of LC PPs are fixed as $\psi_{\mathrm{PP}}=2\pi$ by applied voltage $u=0.98 V$ under room temperature and 633 nm-wavelength-incidence.

- 1. **Phase retardance and switching time of the computing LCs**

Figure S7(a) is measured to show the stability of this phase retardance $\psi_{PP}$ as the ambient temperature changes around the room temperature at 633 nm wavelength incidence (setting $u=0 V$, $u=0.98 V$, and $u=5 V$). Fig. S7(b) presents the dependence of retardance on the voltage at three different temperatures (25℃, 45℃, and 60℃). Figure 7(c) gives a more detailed phase retardance range generated by the LC PP. And Fig. S7(d) shows the electrical switching time of the proposed computing LCs from the higher-voltage to the lower-voltage (i.e., the fall time). It is found that the switching time from 10 V-voltage to 1 V-voltage at room temperature is $310 \mu s$, so as to enable the quick switching and operation. These results further demonstrate the capability of our proposed platform in performing multi-functional spatial differential operations.

**Fig. S6**. Measurement of the spatial spectral transfer function $H\left( k_{x},k_{y} \right)$ and the temperature-dependent phase retardance $\psi_{\mathrm{PP}}$ on computing LCs. (a) Experimental setup. The path 1 and the path 2 realize the optical differential operation along the *x*-direction and the *y*-direction correspondingly, which contributes to the 2D differential operation. PBS, polarizing beam splitter. M, mirror. The remaining components are the same with that in Fig. S1(a). (b) The theoretical (the first row) and experimental (the second row) intensity profiles including the input light, the output light after 1D differential operation along the *x*-direction and the *y*-direction, as well as their 2D results. (c) and (d) are the comparison between the theoretical and the experimental spatial transfer functions $H\left( k_{x},k_{y} \right)$ for ${k_{y}}/{k_{0}}=0$ and ${k_{x}}/{k_{0}}=0$, respectively.

**Fig. S7**. (a) The phase retardance of LC PP as a function of the ambient temperature at 633 nm wavelength incidence. (b) Dependence of retardance on the voltage at three different temperatures. (c) The phase retardance range with the increase of voltage, under 445 nm wavelength and 633 nm wavelength incidences. (d) The switching time of the platform at various temperatures.

**References**

1. A. Yariv and P. Yeh, *Photonics: optical electronics in modern communications.* New York, Oxford university press, 2007.
2. S. Liu, S. Chen, S. Wen, and H. Luo, “Photonic spin Hall effect: fundamentals and emergent applications,” *Opto-Electronic Science*, vol. 1, no. 7, p. 220007, 2022.

1. **Corresponding author: Shizhen Chen**, Key laboratory of Micro-Nano-Optoelectronic Devices of Ministry of Education and Hunan Provincial Key Laboratory of Low-dimensional Structural Physics and Devices, School of Physics and Electronics, Hunan University, Changsha 410082, China; E-mail: chensz@hnu.edu.cn; https://orcid.org/0000-0002-1784-1880

   **Shuoqing Liu, Dandan Zheng, Qiang Yang, Shuangchun Wen, and Hailu Luo**, Key Laboratory of Micro-/Nano-Optoelectronic Devices of Ministry of Education and Hunan Provincial Key Laboratory of Low-dimensional Structural Physics and Devices, School of Physics and Electronics, Hunan University, Changsha 410082, China [↑](#footnote-ref-1)
